# Bioinformatics Unmasks the Maneuverers of Pain Pathways in Acute Kidney Injury

**DOI:** 10.1038/s41598-019-48209-x

**Published:** 2019-08-15

**Authors:** Aprajita Gupta, Sanjeev Puri, Veena Puri

**Affiliations:** 1 Centre for Systems Biology and Bioinformatics, Panjab University, Chandigarh, India; 2 Biotechnology Branch UIET, Panjab University, Chandigarh, India

**Keywords:** Cellular signalling networks, Acute kidney injury

## Abstract

Acute Kidney injury (AKI) is one of the leading health concerns resulting in accumulation of nitrogenous as well as non-nitrogenous wastes in body and characterised by a rapid deterioration in kidney functions. Besides the major toll from the primary insult in the kidney, consequential extra-renal secondary insults endowed with the pathways of inflammatory milieu often complicates the disease outcome. Some of the known symptoms of AKI leading to clinical reporting are fatigue, loss of appetite, headache, nausea, vomiting, and pain in the flanks, wherein proinflammatory cytokines have been strongly implicated in pathogenesis of AKI and neuro-inflammation. Taking in account these clues, we have tried to decode the neuro-inflammation and pain perception phenomenon during the progression of AKI using the pathway integration and biological network strategies. The pathways and networks were generated using bioinformatics software viz. PANTHER, Genomatix and PathVisio to establish the relationship between immune and neuro related pathway in AKI. These observations envisage a neurol-renal axis that is predicted to involve calcium channels in neuro-inflammatory pathway of AKI. These observations, thus, pave a way for a new paradigm in understanding the interplay of neuro-immunological signalling in AKI.

## Introduction

Acute kidney injury (AKI) is a clinical event associated with a rapid loss of kidney function, leading to high morbidity and mortality^1^. Every year about 2 million people die from AKI due to late detection of disease or paucity of effective therapeutic interventions^2^. A number of etiological factors such as ischemia, drugs, exposure to toxins, obstructive nephropathy and sepsis lead to AKI^3^. AKI is manifested by sudden loss of renal functions resulting in accumulation of metabolic waste products within the blood.

The innate and adaptive immune responses, both contribute to the progression of AKI. The tissue damage during AKI occurs in the form of ripped cells, apoptosis, oxygen deprivation as well as stress in the cells^4^. The hallmark features of AKI include inflammatory response, hemodynamic alterations, altered tubule dynamics, cellular ATP depletion, renal cell apoptosis, necrosis and changes in the nervous system^5^. The early response to injury is mediated by damage-associated molecular patterns (DAMPs) which include ATP, interleukin (IL-a), uric acid and high mobility group protein 1 etc. The tissue injury is often accompanied by the accumulation of “inflammatory soup”, which includes endogenous factors, immune cells, cytokines (chiefly interleukin and tumor necrosis factor α (TNF-α)), toll-like receptors, signalling molecules, neurotransmitters, neuropeptides and prostaglandins, that are released from activated non-neural cells, which reside within or infiltrate into the injured area^6^. The spill over of local inflammatory mediators into the circulation excites or lowers the threshold of nociceptive and afferent nerve fibres resulting in activation of the neural axis. This is an adaptive response to acute inflammation which includes cognitive dysfunction, fatigue and sensitivity to pain eliciting a “sickness behaviour”^7^. The chief mechanisms suspected for this response include the inflammatory messengers which signal the CNS through sentinel cells, afferent nerves and neurohormones transporting across the blood–brain barrier (BBB)^8^.

This interaction between immune and nervous system permits rapid homeostatic responses to create inflammation and pain, drifting to mediate protection from injury. However, the neural links of inflammation in AKI have sparsely been explored. In this work we have employed bioinformatics approach to decipher potential molecules that underlie the neuro-immune axis, mainly focusing on the perception of pain in AKI by using software and databases like PANTHER, Genomatix, Target Explorer IPA and KEGG to find the communicators of AKI in the neuro-immune axis.

## Results

### NCBI retrieval of genes

The ‘gene’ search in NCBI integrates information from a wide range of species. The ‘mouse’ restricted search for ‘genes’ with keyword ‘acute kidney injury’ retrieved a list of 663 genes (Supplementary File 1, Sheet 1). The list of genes along with their taxonomy ID, gene ID, map location, start and end position on the genomic accession was retrieved. The list of genes symbols was scooped out from this file to generate a text file.

**Figure 1:**
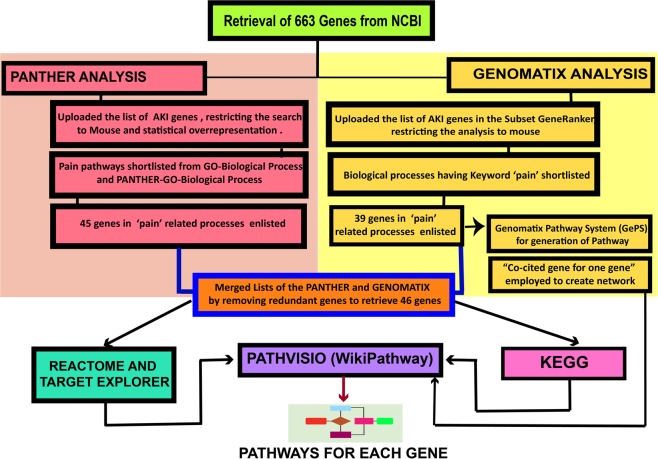
Flowchart of the methodology. Flowchart of the steps followed in finding the pain perception genes and their pathways during AKI. The flowchart shows the software employed and the steps that have been followed in obtaining the desired target genes.

We have employed a ‘two parallel process strategy’ wherein we analysed the gene for functional and ontological characterization via PANTHER and Genomatix bioinformatics tools (Fig. 1).

### PANTHER analysis

**Figure 2:**
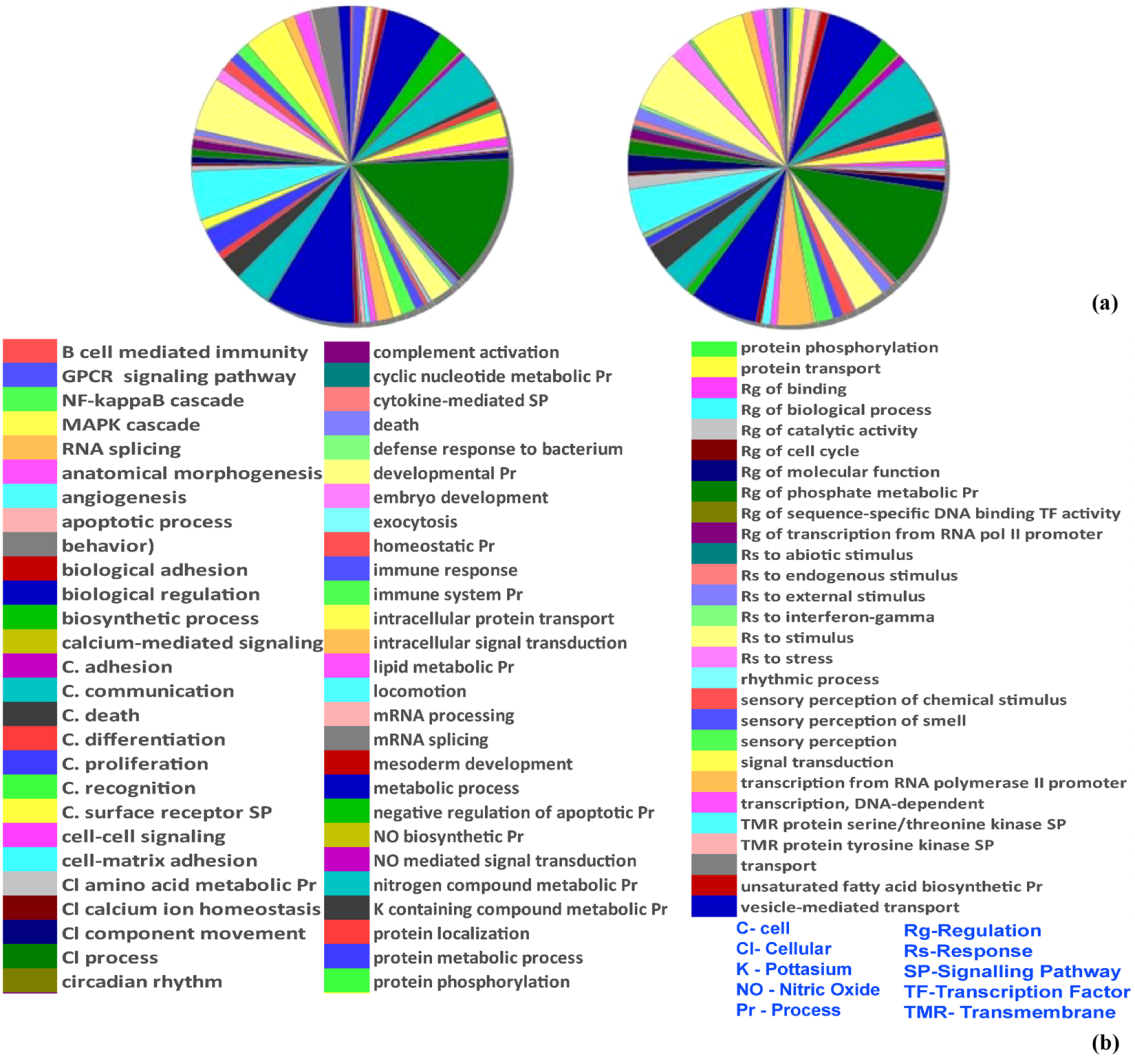
Biological process changes during AKI. (**a**) Pie charts depiction of biological processes in normal mouse (left) vs mouse during AKI (right) as retrieved from PANTHER. The percentage of genes in many processes have significantly modulated when compared to control. (**b**) Legend of the processes depicted in the pie chart.

Retrieved genes were classified functionally for ontological curation by using PANTHER classification system. The annotations of PANTHER datasets were individually analysed to find their association with ‘pain’. Out of four different processes, two biological processes i.e. ‘PANTHER-GO slim biological processes’ and ‘GO-biological process complete’ showed keyword “pain” (Supplementary File 1, Sheet 2, 3). The pie chart of PANTHER-GO slim biological processes depicted the variations in biological processes during AKI (Fig. 2). This data has also been represented in bar graph (Supplementary File 2, Fig A). The graph shows that the highest percentage of genes are involved in the ‘cellular process’, which comprises in the AKI 54.26% of genes as compared to 39.36% of reference mouse genes. Many biological processes showed upsurge in the percentage of AKI genes as compared to the reference. The percentage of genes in the metabolic process (36.62% AKI over 26.99% reference), response to stress (11.36% AKI over 3.53% reference) biological regulation (31.39% AKI over 17.51% reference), cell communication (30.49% AKI over 14.68% reference), and regulation of biological process (24.92% AKI over 14.91% reference) were significantly high. A few biological processes also exhibited a downturn in the percentage of AKI genes when compared to reference. This included G-protein coupled receptor signalling pathways (1.94% AKI over 3.93% reference), protein metabolic process (4.78% AKI over 7.64% reference) and sensory perception (1.64% AKI over 3.98% reference).

Similarly, the graphical analysis of molecular functions (Supplementary File 2, Fig B) showed that the major percentage of genes are included in binding (38.12% AKI over 23.6% reference) and catalytic activity (29% AKI over 19.32% reference). The cellular component analysis shows that the percentage of genes involved in the cytoskeleton decreases in the AKI (0.60% AKI over 2.59% reference) while the maximum percentage of genes are involved in membrane processes (15.84% AKI over 10.71% reference). (Supplementary File 2, Fig C).

During AKI, the percentage of genes in all molecular processes were found to be increased (as compared to reference) except in the antigen binding process. PANTHER GO-Slim Molecular Function having fold enrichment more than 5 times have been enlisted in the Table 1. This generates a list of 11 molecular functions with guanylate cyclase activity having the highest fold enrichment of 13.31 (P < 0.05).

**Table 1:** List of molecular functions from ‘PANTHER-GO molecular functions’ which had increased more than 5-fold in expression during AKI.

PANTHER GO-Slim Molecular Function and their GO number	AKI fold Enrichment	Raw P-value
Guanylate cyclase activity (GO:0004383)	13.31	5.69E-04
Ligand-activated sequence-specific DNA binding RNA polymerase II transcription factor activity (GO:0004879)	11.95	2.38E-10
Tumor necrosis factor receptor binding (GO:0005164)	11.09	4.47E-03
Transmembrane receptor protein tyrosine kinase activity (GO:0004714)	9.02	3.98E-10
Peroxidase activity (GO:0004601)	8.96	3.84E-05
Chemokine activity (GO:0008009)	8.63	4.72E-05
Antioxidant activity (GO:0016209)	8.32	5.02E-06
Cytokine receptor activity (GO:0004896)	7.04	1.86E-06
Growth factor activity (GO:0008083)	6.52	9.84E-06
Phosphoric diester hydrolase activity (GO:0008081)	5.55	1.40E-05
RNA polymerase II transcription factor binding transcription factor activity (GO:0001076)	5.23	2.28E-05

From the list of all biological processes, many processes contained the keyword ‘pain’ which are suggestive of the role of their genes participating and mediating pain during AKI. Hence all the biological processes including the keyword ‘pain’ were filtered (FDR < 0.05) from all GO biological process. This included 6 biological processes as described in Table 2.

**Table 2:** List of GO biological processes involved in pain during AKI. The processes have been aligned according to increasing p-values.

GO Biological Process Complete	AKI Fold Enrichment	Raw P-Value
Detection of temperature stimulus involved in sensory perception of pain	12.94	5.30E-06
Positive regulation of sensory perception of pain	12.48	3.42E-03
Regulation of sensory perception of pain	12.48	3.42E-03
Detection of mechanical stimulus involved in sensory perception of pain	12.26	7.07E-06
Sensory perception of pain	10.32	9.36E-20
Response to pain	5.55	1.29E-03
**PANTHER GO- slim Biological Process**	**AKI Fold Enrichment**	**Raw P-Value**
Sensory perception of pain	4.16	2.34E-01

### Genomatix analysis

The list of genes retrieved from NCBI was uploaded in the Gene Ranker of Genomatix software suite (trial version). The retrieved biological processes containing keyword ‘pain’ were: regulation of sensory perception of pain, response to pain, detection of temperature stimulus involved in sensory perception of pain, detection of mechanical stimulus involved in sensory perception of pain and positive regulation of sensory perception of pain (Supplementary File 1, Sheet 4). The same sets of processes were also obtained from PANTHER with variations in p-values. After removing the redundant genes, we retrieved a list of 39 genes from Genomatix.

### Merging the persistent Neuro-inflammatory genes

The list of genes from PANTHER and Genomatix containing 45 and 39 genes respectively were merged to generate a combined list. Most of the genes overlapped in the two outputs and list of 46 genes was obtained. There were 7 genes viz Ace, Adcyap1, Cxcl12, Cxcr4, grk2, Mapk1 and Scnn1a, which exclusively made presence in PANTHER annotation list but not in Genomatix (Table 3).

**Table 3:** The combined list of genes obtained from PANTHER-GO and Genomatix, that participate in pain pathways during AKI.

List of genes common for the two databases				List of genes exclusive to Panther
Adora1	Ednrb	Mapk3	Ptafr	Ace
Alox5	Ephx2	Mme	Ptgs1	Adcyap1
Aqp1	F2r	Mtor	Ptgs2	Cxcl12
Bdkrb1	Fcgr4	Ngf	Rtn4	Cxcr4
Calca	Fgfr1	Ntrk1	Slc9a1	Grk2
Ccr2	Fyn	P2rx4	Thbs1	Mapk1
Cnr2	Grin1	P2rx7	Tlr4	Scnn1a
COX2	Il10	P2ry1	Tnf	
Ctss	Il18	P2ry2	Trpv1	
Edn1	Il1a	Prkcg		

Hence, from a list of 663 genes that tenaciously participate in AKI, we narrowed down our search to 46 most putative inflammation-pain genes. PANTHER considerd COX and Ptgs2 as one entity while Genomatix enlisted only ptgs2 and does not enlist COX (Supplementary File 1, Sheet 3). In order to avoid any chance of missing of the genes during text mining approach, we have included both the gene names Cox and Ptgs2 making the merged list as 46 instead of 45.

Each of the gene was analysed in Genomatix and its respective interaction network, disease related network, cell level signalling and gene interaction with small molecules was retrieved along with their co-cited genes (Supplementary File 1, Sheet 5). We further analysed on genes participating in inflammation as well as in pain pathways. These interactions indirectly or directly may affect each other’s function and contribute in AKI pathology. From the list of 46 genes, we depict the probable eleven routes of 12 most eminent genes through which AKI mediates pain-inflammation signals. Combining all these gene data, hypotheses have been framed, regarding the neuropathic pain pathways during AKI. We have merged the inflammation and pain perception genes to design plausible pathways depicting the routes through which AKI might lead to perception of pain along with inflammation.

The probabilistic models of these genes have been categorised into two categories:

(i) Pro-Neuroinflammatory and (ii) Anti-Neuroinflammatory.

**(A) Pro-Neuroinflammatory genes**

**A.1: Alox**

**Figure 3:**
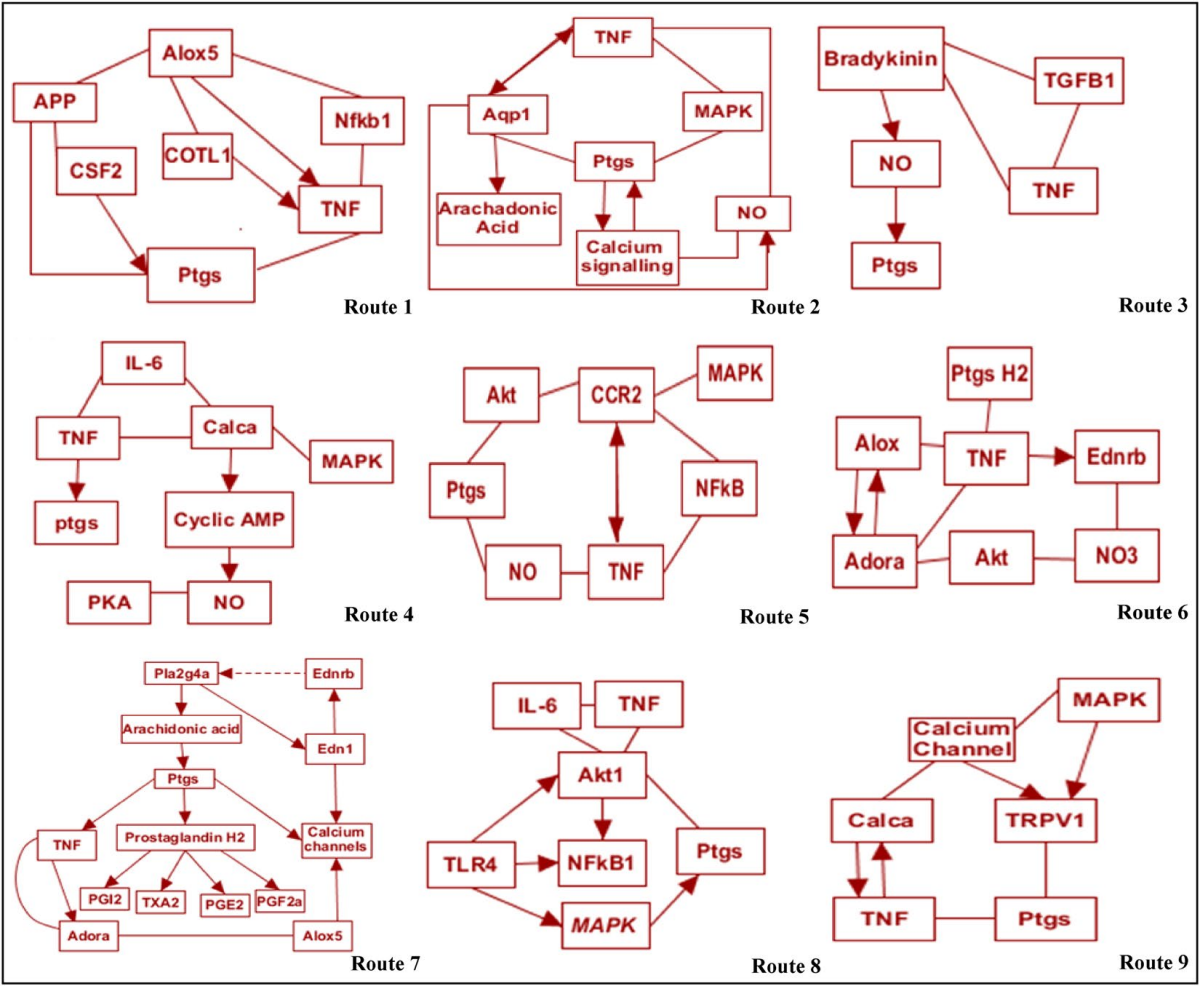
Bioinformatics based pathways of pro-neuroinflammation derived pain mediation during AKI. Sugeestive pathways of the putative pro-neuroinflammatory genes that mediate pain during AKI have been shown. The genes follow different routes and interaction pathways to cause neuroinflammation as shown in the routes.

This gene stands for arachidonate 5-lipoxygenase. It encodes a member of the lipoxygenase gene family playing a role in synthesis of leukotrienes from arachidonic acid. The list of over-represented diseases and mesh diseases generated by Genomatix (Supplementary File 1, Sheet 6 and 7) shows that Alox participates in inflammation, AKI, glomerulonephritis and nervous system autoimmune disease. The Genomatix pathways also show that Alox interacts with TNF and Ptgs, reflecting its mediation in pain as well as inflammation. It reticulates through Trp53, App, Ptgs2, MAPK1, and TNF which play a role in various metastasis, neurodegenerative disorders, pain and inflammation respectively. The signal transduction pathway association (Supplementary File 1, Sheet 8) shows that Alox association is mediated via angiotensin, interleukin and TNF pathway. (Route 1, Fig. 3).

**A.2: Aqp1 (Aquaporin 1)**

Aqp1 gene encodes a small integral membrane protein with six spanning layers functioning as a water channel protein. The list of over-represented diseases as well as list of mesh-diseases generated by Genomatix (Supplementary File 1, Sheet 6 and 7) show that Aqp1 (along with other genes) participates in diseases such AKI, CNS disorder, inflammatory disorders and autoimmune diseases. Aqp1 reticulates and interacts with genes such as TNF, Vegf, Agt, NO and MAPK. Target explorer and signal transduction pathway association (Supplementary File 1, Sheet 8) by Genomatix indicate that Aqp1 is regulated by hypoxia inducible factor 1 network, GPCR pathway signalling the nerves and modulating the calcium channel regulation. The aquaporins also affect the expression of Alox gene. (Route 2, Fig. 3).

**A.3: Bdkrb1 (Bradykinin receptor)**

Bdkrb1 gene encodes a receptor for bradykinin. The 9 aa bradykinin peptide elicits multiple responses including vasodilation, smooth muscle spasm, edema and pain fibre stimulation. Bradykinin receptor associates with G proteins to stimulate phosphatidyl-inositol-calcium pathway. The analysis of over-represented diseases and mesh diseases generated by Genomatix (Supplementary File 1, Sheet 6 and 7) reveal role of Bdkrb1 in inflammatory pain, neuropathy, and chronic inflammation and CNS disorder. The Genomatix pathways and signal transduction genes list (Supplementary File 1, Sheet 8) shows that Bdkrb1 interacts with TNF and Ptgs, reflecting its putative role in inflammation as well as pain sensation. It signals through PKC, ERK and MAPK pathways ultimately modulating calcium pathways (Route 3, Fig. 3).

**A.4: Calca (Calcitonin)**

Calcitonin gene (Calca) is involved in calcium regulation and regulates phosphorus metabolism. It interacts with MAPK, TNF and IL-6 signalling. The list of over-represented diseases and list of mesh-diseases generated by Genomatix (Supplementary File 1, Sheet 6 and 7) reveal the role of Calca in vasodilation, inflammation, CNS sensitization and chronic pain. The signal transduction pathway association (Supplementary File 1, Sheet 8) shows that Calca networks through nitric oxide synthase, protein kinase C and guanylate cyclase. The Genomatix pathway shows that Calca interacts with TNF, IL6, Ptgs2, and Akt hence mediates inflammation, and pain (Route 4, Fig. 3).

**A.5: Ccr2 (C-C chemokine receptor type 2)**

Ccr2, C-C chemokine receptor type 2, a cognate receptor for MCP-1, is a seven-transmembrane-spanning chemokine receptor located in the chemokine receptor gene cluster region. It has been shown through Genomatix (Supplementary File 1, Sheet 6 and 7) that Ccr2 participates in various pathologies including AKI, inflammatory disorders, vascular diseases and inflammatory pain. The pathways generated in Genomatix, the signal transduction pathway association (Supplementary File 1, Sheet 8) and target explorer narrate that Ccr2 schmooses via TNF, MAPK1, IL-6, Agt, Rela and IL-10 which triggers ptgs2 (Route 5, Fig. 3).

**A.6: Ednrb (endothelin receptor type B)**

Endothelin (ET)-1 is a potent vasoconstrictor peptide with pro-inflammatory, mitogenic, and pro-fibrotic properties in both normal renal physiology and pathology. The endothelin receptor type B (EDNRB) has been known to play a role in key events in progressive renal injury like inflammation, pain, and cell infiltration (Supplementary File 1, Sheet 6 and 7). The pathways generated by Genomatix (Supplementary File 1, Sheet 8) reveal that signalling pathway responsible for this effect is thought to occur via Gs. The IP3 (Inositol trisphosphate) production through Gαq, ostensibly activates Phospholipase C. This mediate a rise in intracellular calcium along a pathway which might lead to β-endorphin release (Route 6, Fig. 3).

**A.7: Ptgs (Prostaglandin Synthase)**

Prostaglandins are lipid mediators implicated in a variety of physiological and pathophysiological processes in the kidney, including renal hemodynamic, body water and sodium balance, and the inflammatory injury characteristic in multiple renal disease (Supplementary File 1, Sheet 6 and 7). The Genomatix files show that ptgs1/2 regulation occurs in inflammatory disorders, sensitization and necrosis. The gene is a member of the G-protein coupled receptor family (Supplementary File 1, Sheet 8). It interacts with TNF and inflammatory mediators like IL-6. The NOs signalling and Agt regulation occurs in ptgs1/2 regulation. (Route 7, Fig. 3).

**A.8: TLR4 (Toll-like receptors 4)**

Activation of innate immune response during AKI through TLRs contribute to brain-kidney interactions. TLR4 has been known to contribute to neuronal injury during ischemia-reperfusion injuries as well as regulation of cytokines such as TNF, IL-6, iNOS and IFN-g.

It networks in inflammatory pain, nervous system sensitization and chronic pain (Supplementary File 1, Sheet 6 and 7) and signal transduction pathways of many immune pathways (Supplementary File 1, Sheet 8), (Route 8, Fig. 3).

**A.9: TNF (Tumor Necrosis Factor** *)*

TNF is a hallmark of AKI. it is involved in the regulation of a wide spectrum of biological processes including pain, differentiation, inflammatory pain, renal interstitial fibrosis cell invasion, apoptosis, lipid metabolism and coagulation (Supplementary File 1, Sheet 6 and 7). It is regulated by NFAT, ILs, and mediates inflammatory effect via NFκB, p53 and HIF (Supplementary File 1, Sheet 8), (Route 8, Fig. 3).

**A.10: TRPV1 (Transient Receptor Potential Cation Channel Subfamily V Member 1)**

TRPV1 gene are expressed by nociceptor neurons and are involved in pathogenesis of pain. The data list generated by Genomatix (Supplementary File 1, Sheet 6 and 7) show that TRPV1 (along with other genes) participates in diseases such AKI, CNS disorder, inflammatory disorders, central nervous system sensitization and autoimmune diseases. TRPV1 reticulates and interacts with genes such as TNF, Ptgs, Calca, Agt, NO and MAPK. TRP channels such as TRPV1 interacts with TNF, NGF and also signals MAPK, GPCR mediated PKC pathway (Supplementary File 1, Sheet 8). TRPV1 channels also interacts with the neuropeptides CGRP (Route 9, Fig. 3).

**(B) Anti-Neuroinflammatory**.

**B.1: Adora**

**Figure 4:**
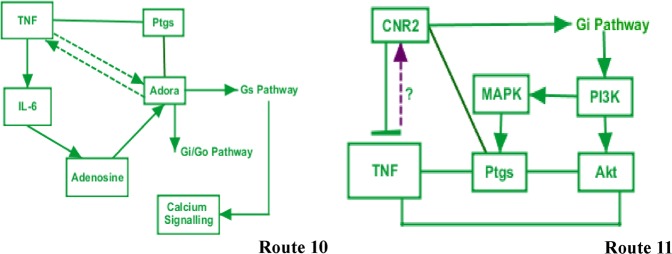
Bioinformatics pathways of anti-neuroinflammation derived pain mediation during AKI. Suggestive pathways of the putative participating genes that play a role in inhibition or reduction of the neuroinflammation during AKI. Their proable course of mediation and interactions to achieve their path have been shown here.

The protein encoded by gene Adora is an adenosine receptor belonging to G-protein coupled receptor 1 family. The data list generated by Genomatix (Supplementary File 1, Sheet 6 and 7) show that Adora participates in a number of diseases such as AKI, renal insufficiency, inflammatory disorders, diabetic nephropathy, brain ischemia, renal interstitial fibrosis and central nervous system sensitization. For such roles, Adora reticulates and interacts with genes such as TNF, IL-6, NFκB, Akt-1 and MAPK among many. TNF further opens its wings to give flight to genes such Casp3, Tgf and IL10. NFκB mediated by TNF also mediates inflammatory processions (Supplementary File 1, Sheet 8) (Route 10, Fig. 4).

**B.2: Cnr2**

Cnr2 (cannabinoid receptor 2) is a functionally active endocannabinoid system is present within the kidney. The cannabinoid receptor type 2 (Cnr2) is expressed by inflammatory cells as well as podocytes. Its presence has been observed by Genomatix in pathologies of AKI, pain, fibrosis, inflammatory pain, CNS disorder and sensitization (Supplementary File 1, Sheet 6 and 7). Cnr2 mediates its network by interacting with NOS, IL-6, Ptgs2, MAPK1, Akt1, TNF and NFκB and signalling through many pathways including interleukin pathway (Supplementary File 1, Sheet 8) (Route 11, Fig. 4).

## Discussion

**Figure 5:**
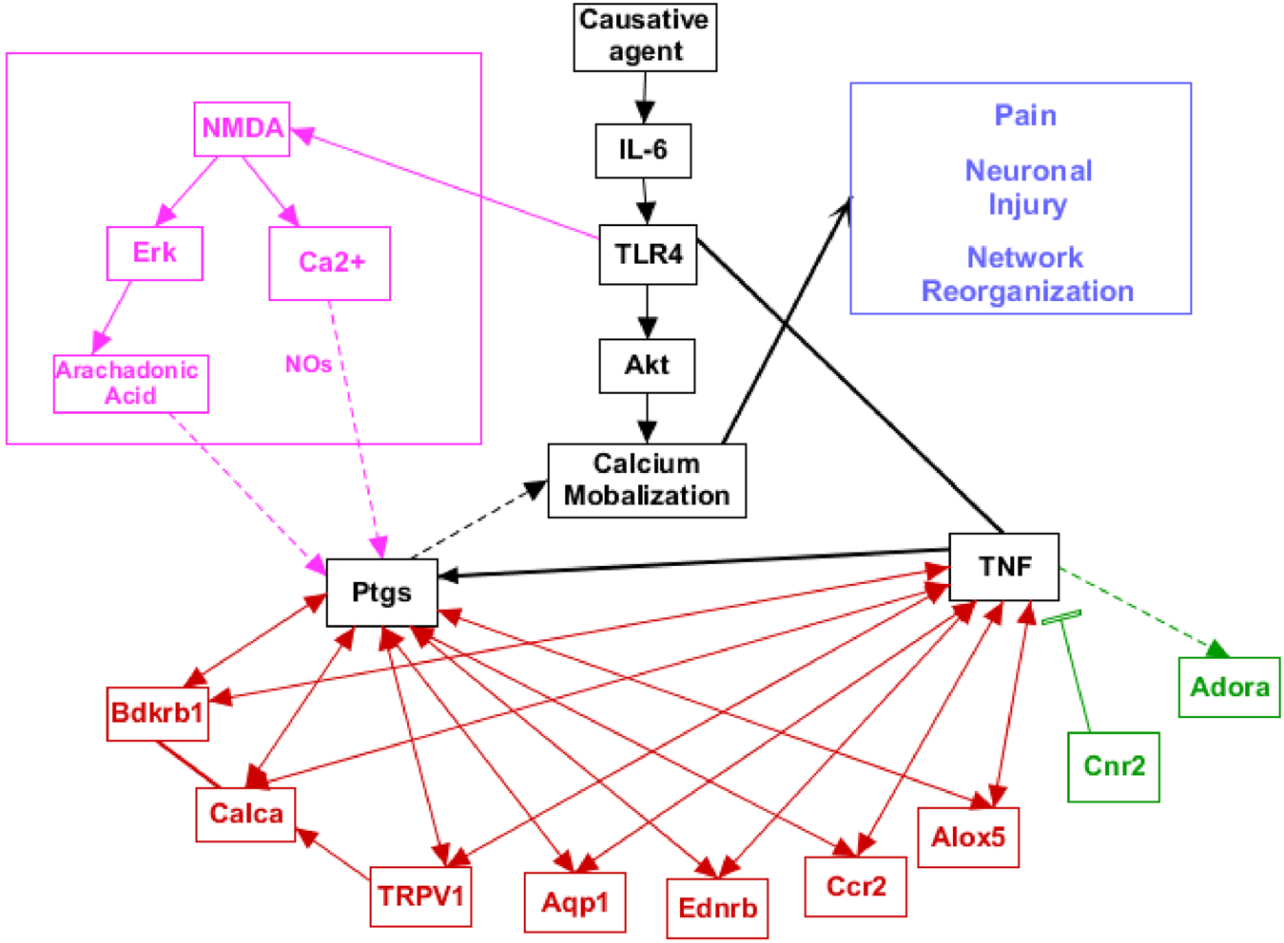
Role and interaction of putative genes during AKI. The interplay of genes with respect to each other has been shown in the diagram. The genes in red are pro-neuroinflammatory and the ones in green are anti- neuroinflammatory. The genes shown in black have a mixed role depending upon the genes in the surroundings.

AKI is associated with pathways of inflammatory milieu, which gradually lead to stress, apoptosis, vascularity modulation, hormonal change, intonation in the nervous system, disruption of homeostatic balance, and damage or failure of other organs^1^. This integration triggers an array of other physiological events associated with multiple genes and proteins of different organs, systems and pathways. The kidneys have the ability to repair and regenerate themselves after AKI to a certain extent, yet a complete and successful treatment of patients with AKI remains one of the greatest challenges facing nephrology today^9^. The patients of AKI show cardinal symptoms of pain such as hyperalgesia, and apparent spontaneous pain in flank. Sickness and depressive behaviours^10^ have also been reported in AKI. Several inflammatory mechanisms have been implicated in sensitization of nociceptors, wherein cytokines are thought to play a role^11^. A potential common mechanism for inflammation, depression and pain is most likely to be linked to kidney-brain signalling. We have followed a de-novo pathway mapping approach by bioinformatics intervention to elucidate significant changes, major pathways and signalling cascades in order to uncover potential targets of pro and anti-neuroinflammation during AKI in a molecular mechanistic way. At an initial finding, a list of 663 genes reported so far in modulation of AKI in *Mus musculus* was retrieved from NCBI. This list has an apparent complexity because a large number of kidney cell types participate in various processes such as apoptosis, inflammation, stress and neuron signalling, as supported by literature reports. Many signalling molecules are activated in more than one form, inducing AKI via multiple entry points or provocations. Moreover, each gene has multiple context dependent functions which reticulate and modulate the behaviour of other genes. Such an approach has been deciphered by bioinformatics approach and a composite network of all pathways has been elaborated in Fig. 5.

We have taken the lead from our previous studies^11^ where we have shown the role of NFκB/RelA and ROS family in AKI. It was shown that TNF is a key player in the pathophysiology of AKI. Our pathways elucidation shows a direct interaction of TNF with IL-1β. The literature reports bring forward the role of IL-1β in transcription induction of cyclooxygenase enzyme, thereby aiding in the production of prostaglandin, responsible for pain and fever. TNF has been known to affect domains associated with depression, sickness behaviour^12^, neurotransmitter metabolism, synaptic plasticity and neuroendocrine function^13^ correlating with higher neuropathic pain^12^. The increased level of TNF lead to reduction in hippocampal volumes linked to depressive behaviours^14^. TNF is known to mediate the mechanism of neuropathic pain through glial systems by involving G-protein coupled receptor, TNF converting enzyme, production of IL-1, IL-6, nitric oxide and ATP^15^, most of these have also been reflected in our pathway analysis. TNF signals via MAPKs^16^, JNK pathway to the leukocyte recruitment, cytokine staffing, release of vascular effect genes such as VEGF and EDN1, and renin activation leading to RAAS modulation^17^. Literature reports strongly suggest that these processes are mediated by the TLR. Among these receptors, TLR4 contributes in neuronal injury as well as inflammation. It raises the possibility that inflammasomes involving TNF and TLR may stimulate the neural circuits^18^.

Cytokines like interleukins or tumor necrosis factor (TNF) produced by locally galvanized immune cells are pivotal in this communication from immune system to the nervous system^19^. The activation of TLR, NFκB, IL and cytokines recruit macrophages to lead a positive cycle of inflammation, sensitizing nociceptors. In the cell bodies of dorsal root ganglion, the cell bodies of nociceptors interact with inflammatory mediators, to sensitize neuron signalling and increase nociceptive response^20^ to elevate IL, prostaglandins, and their association with MAPK^21^. Parallel to these reports we have obtained pathways signalling the similar machanistic route in AKI.

Kidneys are industrial units for the production of prostaglandins. The mediators of neuro-inflammation such as IL-1β, TNF and nitric oxide induce production of prostaglandins via cyclooxygenase (COX)^22^. Prostaglandins are produced in medulla and route via the loop of Henle to the macula densa^23^. The stimulation of receptor 1 of prostaglandin (EP1) leads to activation of protein kinase C to upsurge in the intracellular calcium. EP2 and EP4 receptors are mediated through the Gs protein leading to an increase in cAMP. The DRG neurons and the nerve terminals in renal pelvis contain EP4 receptors and CGRP receptors. In renal pelvic wall, prostaglandin E2 (PGE2) stimulates EP4 receptor and thereby activating mechanosensory nerves located on or adjacent to the sensory nerve fibres^24^. Under pathological conditions, PGE2 functions through GPCR binding receptors to mediate the renal injury. Our results show that Ptgs participates in pain and inflammation pathways during AKI and interacts with TNF, Alox5, and IL-6 and signals via calcium dependent pathways. Literature supports that in the kidney, the receptors EP4 and EP1 mediate glomerular injuries^25^. Increased levels of PGE2 upregulates expression of the EP4 receptor subtype in rat sensory dorsal root ganglion (DRG) neurons^26^. The EP4 receptor mRNA contribute to the regulation of glomerular hemodynamic and renin release functions via a calcium-dependent signalling pathway^27^.

Most of the afferent renal nerves which contain substance P and calcitonin gene-related peptide (CGRP) are situated in the renal pelvic wall^28^. The CGRP (calca) also increases the oxidative stress and induces apoptosis in the ischemia reperfused kidneys^29^. Our pathways elucidation show that Calca interacts with TRPV1 and thereby its interaction with TNF is mediated. CGRP affects the central nervous system via the afferent nerve fibres, thereby causing a modulation in release of cytokines and activation of afferent or efferent renal nerves; creating a loop in local inflammation and injury^30^. This signalling is mediated by MAPK and NO interaction which serve as proliferation points of calcium signalling pathway as suggested by our computational approach and supported by literature^31^. Pathways like NO and MAPK mediate the networking for both the genes and serves as common linking point for bradykinin and Calca. The active NFκB in turn encourages the expression of a diverse array of genes such as 5-LOX and COX-2^32^. Parallelly ROS-induces ERK1/2 activation and its stimulation leads to activation of NFκB and, consequently promoted AKI. Alox5 (also called 5*-*LOX)  is abundantly expressed in hematopoietic cells and immune cells. Their role has been found in neuronal abnormalities, immune modulations, ischemia conditions and skin diseases. The Ca^2+^ regulates the expression of the Alox5 gene. Alox5 seems to be closely involved in this process of neutrophilic inflammation^33^. Alox5 is one of the NF-κB targets that generates ROS during arachidonic acid metabolism^34^. The Genomatix pathways show that Alox5 interacts with TNF and Ptgs, reflecting its mediation in pain as well as inflammation.

The kidney is central to many homeostatic mechanisms in the body, and as AKI progresses, dysregulations occur in various locations. Aquaporins (AQPs) are integral membrane proteins known to be expressed in the kidneys and play an important role in the renal water handling. This protein permits passive transport of water along an osmotic gradient. In normal brain it facilitates the secretion of cerebrospinal fluid restricting itself to the choroid plexus. Aqp1 is important in avoiding negative fluid balance during AKI and for disorders involving imbalance in fluid movement. Aqp1 interacts with TNF and Ptgs via mediators such as HIF and NO respectively^35,36^ to manage homeostasis in AKI.

The cytokines and downstream mediators of the inflammatory surge can also channelize functions in CNS by acting directly or indirectly on neurons, and affecting their excitability threshold at cellular and network levels. Recent studies have revealed that Ccr2 signalling is involved in tubulointerstitial damage, human crescentic glomerulonephritis and pathogenesis of renal ischemia-reperfusion injury through infiltration and activation of macrophages^37^. Our pathways suggest that Ccr2 signals via Agt, Rela, Nos and TNF^38^. Many reports support these findings that MAPK, p38/MK-2 signalling cascade has fundamental roles in mediating Ccr2 signalling and help in neuroinflammation and modulating neuronal and glial survival^39^. Ccr2 signalling mediates these kidney diseases by Agt, NFκB, Notch and Rela regulations. It has been shown that during neuropathic pain caused by peripheral nerve injury, MCP-1 and Ccr2 expression increases in the DRG. The simultaneously coordinated inflammation and neuronal excitability by MCP-1/CCR2 signalling contribute to the pro-analgesic actions of the chemokine^40^.

Among many pathways that aid in progression of AKI, the endothelin receptor subtypes, termed ET-A and ET-B, have been known to play a vital role. The receptor for endothelin (ETA, ETB), are widely distributed within the human kidney, the ETB receptors are more in number (ETB-to-ETA ratio 2:1). The ET-A receptors mediate vasoconstriction and the ET-B receptors mediate the release of prostaglandins and NO^41^. The ETB promotes renal inflammation, oxidative stress, vascular shear stress and hypoxia^42^. These receptors also induce synthesis, release of prostaglandin E2 and also mediate release of β-endorphin hence thereby producing a local analgesic effect which further activates DAG and Ca^2+^ via G signalling pathway. This molecular pattern has been paved in our analysis suggesting the interaction of Ednrb with HIF, Agt, TNF and MAPK1 to mediate similar actions as validated in other systems^41^.

The dynamics of ion selectivity such as Ca^2+^ is monitored by vanilloid Transient receptor 1 (TRPV1) channels which are well distributed in many organs including kidneys. The role of TRPV1 has been implicated in AKI as well as in our current pathways. Various reports show the involvement of TRPV1 channels, ensuing CGRP and substance P rise during AKI^43^. Parallel to this observation is the role of Bradykinin receptors who promote synthesis of prostaglandins^44^.

One of the genes shortlisted in our studies is Cnr2. The role of the endocannabinoid system in the kidney after activation of G protein–coupled receptors (GPCRs) via cannabinoid receptors is emerging as an important response system after injury stimuli^45^ Cnr2 is associated with immune regulation and function. Its role has been investigated in neurological disorders associated neuroinflammation and neuropsychiatric disturbances. Cnr2 activation in macrophages protects from hepatic inflammation through an autophagy-dependent pathway, and also mediates a conversation between the nervous and immune systems and plays a role in maintaining immune homeostasis in the gut/pancreas^46^. It is observed that during neuroinflammation, the Cnr2 expression up-regulated in the context of amyloid-triggered neuroinflammation^47^. Cnr2 protects the kidney damage by reducing inflammation and oxidative/nitrosative stress by limiting the endothelial inflammatory response and inflammatory cell adhesion during reperfusion injury^48^. Similar to present analysis, other reports have also suggested, that Cnr receptor can interact with Gaq to promote activation of phospholipase C. This can further trigger Akt crucially involved in promoting cell survival via the antiapoptotic proteins such as Bcl-2^49^.

The pathophysiology of AKI prescribes the specific Adenosine receptor activation that is required to produce renal protection^50^. The activation of Adora 1 decreases inflammation and apoptosis; and modulates the metabolic action, in renal tubules and endothelial cells to prevent kidneys from injury progression^51^. Adenosine receptors have ability to limit the TNF production; the A_1_ receptor agonists can prevent inflammation-mediated organ injury in animal models^52^. Interestingly, it has been suggested that the enhancement of adenosine A_1_ receptor expression might efficiently induce adenosine A_1_ receptor-mediated neuroprotection^53^. Another study has suggested that increasing the adenosine receptors A_1_ might lead to an efficient neuroprotection by down regulation of post synaptic NMDA receptor-mediated currents and by inhibition of glutamate release from the presynaptic terminal^54^. The stimulation of A_1_ and A_3_ receptors can also elicit the release of calcium ions from intracellular stores. This process is mediated by IL-6, activating VEGF^55^ and leaving a beneficial impact on neuronal survival. Considering these protective roles of A_1_ receptor, it is being targeted as an avenue for the development of new therapeutic methods against neuropathic pain. The Adora 1 has a biphasic role in the development of AKI at different time points in ischaemia rats. It is argued that AR1 activation sequentially induces adenosine A_2A_ receptor expression and by this promotes the resolution of inflammation^56^. The fibroblast in kidneys also express a functional AR_1_ receptor that inhibits cAMP upon stimulation. Adora 1 works via G signalling pathway to activate PLC, MAPK pathway generating both Pro and Anti-inflammatory pathways^54^.

The processes of renin release and glomerular filtration rate (GFR) are regulated by adenosine in the kidneys^57^. Adenosine is physiologically present at low levels in interstitial fluids of normal functioning tissues, but in response to pathological conditions such as hypoxia inflammation or ischaemia the level of adenosine quickly increases. This alarm the danger signal in the body and aims to restore tissue homeostasis. A signal beyond the AKI leads to promotion of pathways involved in wound-healing and repair pathways^58^. Adenosine increases the blood flow to the tissues as per the metabolic demand. Most of the blood vessels respond to adenosine by dilation, except the afferent arterioles of the kidney where adenosine A_1_ receptors (Adora1) mediate contraction in response to adenosine coming from interstitium^59^. This forms the well-known tubule-glomerular feedback circuit which causes superfluous adenosine formation in response to excess transport work in the kidney tubules. The surplus formation of adenosine is taken to glomeruli where it reduces the tubular transport by decreasing the blood flow^60^. The activation of A_1_ receptor can directly activate K^+^ channels and inhibit Ca^2+^ channels. Genomatix analysis shows that mTOR, P13 and GSK3β are in direct interaction with Adora1.

It appears persuasively evident that this data is merging to inflammation and pain via MAPK, Akt, GPCR and Ca^2+^ pathways leading to deleterious events seen in AKI. Collectively, based on the present data, we could hypothesize that CGRP pathway which is responsible in conjunction with sustained RAAS activation and ultimately vasodilation as well as pain may be a major contributor to these events. The activation of CGRP and its cross talk with TNF mediated by TPRV1 receptors in tubular cells or podocytes promotes oxidative stress, mobilization of Ca^2+^-signalling and adenylate cyclase cascades and interlinking ROS pathways leading to apoptotic cell death similar to the neurological environment. Additionally, aquaporins may mediate channel modulation resulting in modulation of calcium channels leading to cell death. Remarkably aquaporins interact with eNOS to aid in recruitment the pro-inflammatory factors. The genes associated with AKI screened to construct the pathways networks and subsequent bioinformatics analyses could more effectively provide the plausible AKI drug targets, and specific signalling networks for reactive neuro-inflammation from AKI. With a constantly expanding repertoire of techniques, this new information on genes of AKI will definitely generate tremendous possibilities for laboratory examinations and it will contribute for advancement of current approaches to therapeutics and monitor the progression of AKI.

## Methodology

### Retrieval of mouse AKI genes from the NCBI

List of all the ‘genes’ in NCBI with keyword ‘Acute kidney injury’ for mouse were downloaded from NCBI and stored in the form of a text file. This was followed by pathway analysis by a dual approach. The data analysis has been strengthened by employing at least two databases and software. The data analysis strategy designed for the study has been elaborated in Fig. 1.

### PANTHER analysis

The gene list was curated using PANTHER (Protein Analysis Through Evolutionary Relationships) classification system, version 11.1 (http://www.pantherdb.org). The PANTHER compares the data of selected genes with reference organism (in this case Mus musculus) and classifies genes either on the basis of their functions in case of experimental evidence or by using evolutionary relationships to predict functions during the lack of direct experimental evidence. PANTHER provides the data based on the merger of two primary sources viz PANTHER and the Gene Ontology (GO). We have used the version 11.1 in the current study. Since, PANTHER provides more up-to-date annotation data (updated monthly), hence the representation of results will update with every revised version of database.

PANTHER provides nine annotation datasets (based on PANTHER and GO) viz PANTHER Pathways, PANTHER GO-slim molecular function, PANTHER GO-slim Biological process, PANTHER GO-slim cellular components, PANTHER protein class, GO molecular function complete, GO biological Process complete, GO cellular component complete and Reactome Pathways. The list of all the annotation datasets was exported and analysed. To assess the statistical enrichment (using default settings) from our dataset, relative to the global set of mouse genes, a Fisher’s Exact with FDR multiple test correction within the PANTHER system was applied. (percentage of gene list in the category is calculated for each testing list as: No. of genes for the category/No. of total genes in the list * 100). Various annotation data sets were applied on the uploaded files and their annotation scores were obtained. The scores include fold enrichment of a gene (which shows statistically over- and under-represented biological processes among the genes in each cluster), Raw P value and false discovery rates (FDRs). Similarly, the molecular functions significantly changed in AKI were selected using high-stringency of p-values < 0.05 and fold changes >2. All these files were analysed for annotation information followed by shortlisting of the pathways or processes which included the keyword ‘pain’.

### Genomatix analysis

We used Genomatix software (trial version ElDorado 12-2017) (https://www.genomatix.de). The Gene Ranker subset of Genomatix was used to generate the list of the curated genes. The Genomatix provides a literature-based output of genes for biological processes, gene ranker list of cellular components, diseases, co-cited genes etc. We downloaded all the lists and looked for all the gene categories with key word ‘pain’ in biological pathways. All the pathways and the list of corresponding genes were downloaded. Just like the PANTHER analysis, the keyword ‘pain’ was manually searched.

### Retrieval of the pathways for the genes

Initial pathway study was done using Genomatix pathways, KEGG (Release 88.2) and Reactome. The De-novo pathway maps were constructed by extended manual literature searches in PathVisio (version 3.3.0). The pathways were drawn using WikiPathway maps, where metabolic and signalling cascades were identified. Gene activation cascades were put together by manual literature mining and use of on-line resources such as GeneCards, Target pathway of IPA by Qiagen and Genomatix. The delineated pathways were then combined into plausible signalling cascades and initial sub-models were manually assembled using the PathVisio software. A final model was established after several re-iterations and literature cross-checks.

## Supplementary information

Supplementary File 1

Supplementary File 2
