# Supplementary material for: Bioinformatics Unmasks the Maneuverers of Pain Pathways in Acute Kidney Injury
Source: Sci Rep. 2019 Aug 15;9:11872. doi: 10.1038/s41598-019-48209-x (PMC6695489; doi:10.1038/s41598-019-48209-x)
Supplement: Supplementary file 2 [file 41598_2019_48209_MOESM2_ESM.pdf]

Bioinformatics Unmasks the Maneuverers of Pain Pathways in Acute Kidney Injury

Aprajita Gupta[1], Sanjeev Puri[2], Veena Puri[1]*.

1. Centre for Systems Biology and Bioinformatics, Panjab University, Chandigarh, India.
2. Biotechnology Branch UIET, Panjab University, Chandigarh, India.

vpuri1825@pu.ac.in

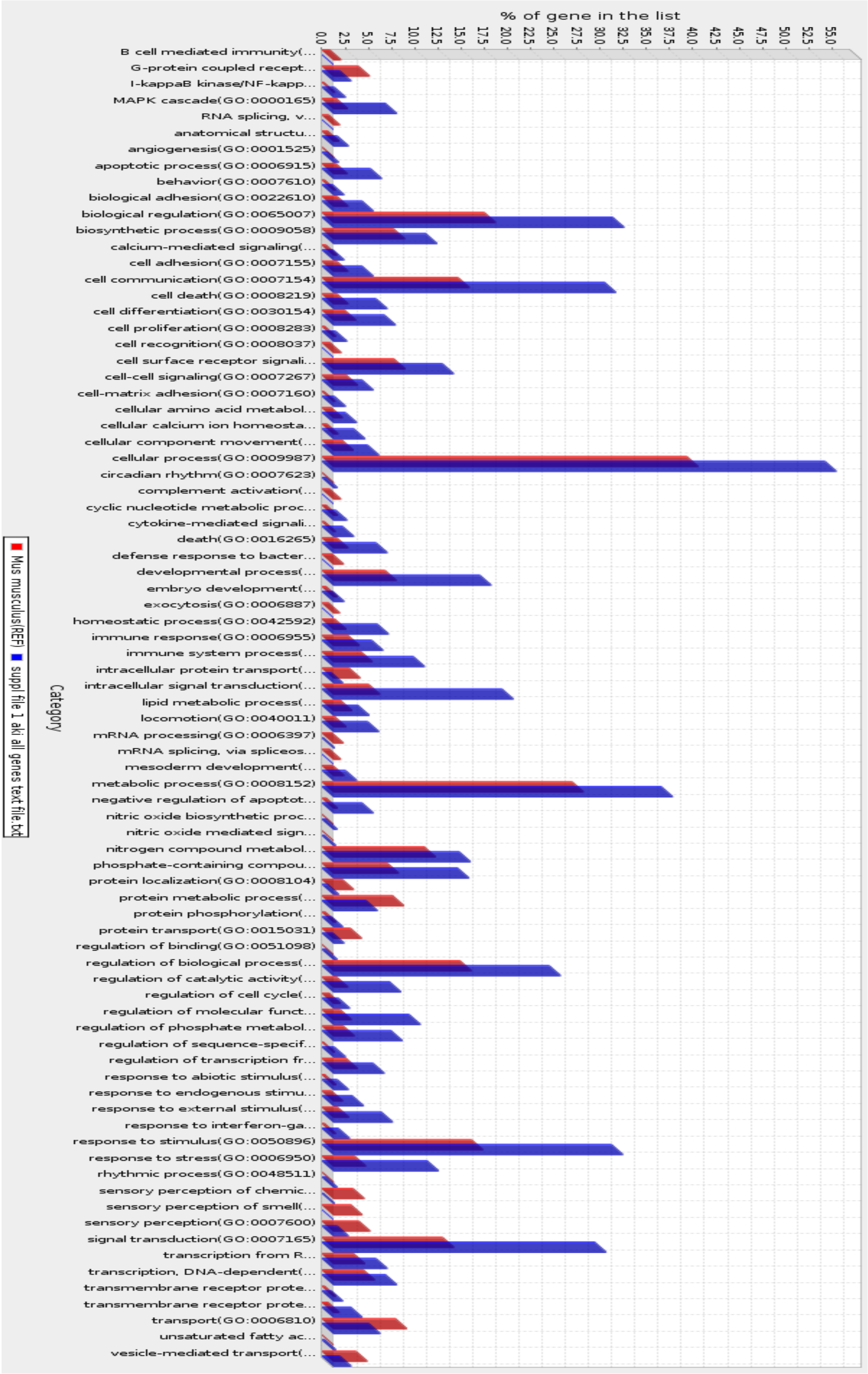

Fig A: Bar chart generated in PANTHER to compare the gene count of AKI genes with the reference mouse genes during various biological processes.

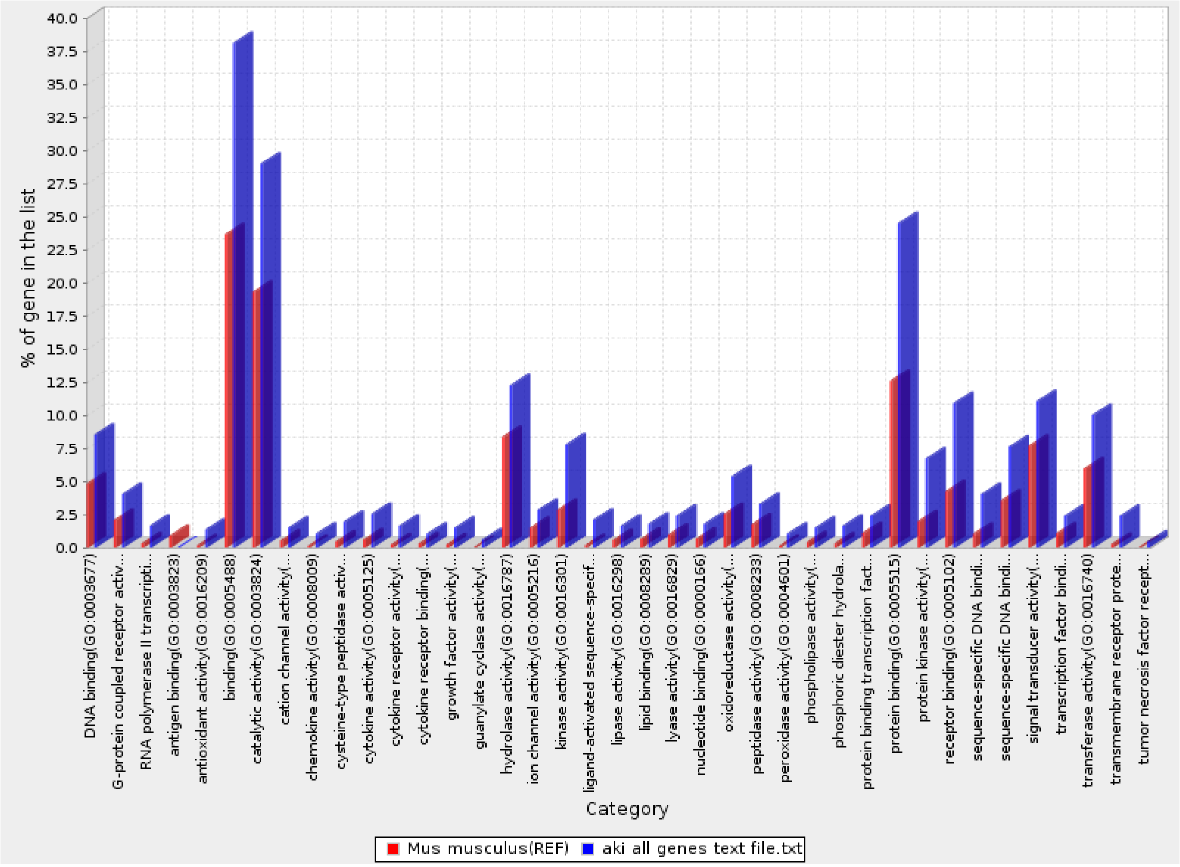

Fig B: Bar chart generated in PANTHER to compare the gene count of AKI genes with the reference mouse genes during various molecular functions.

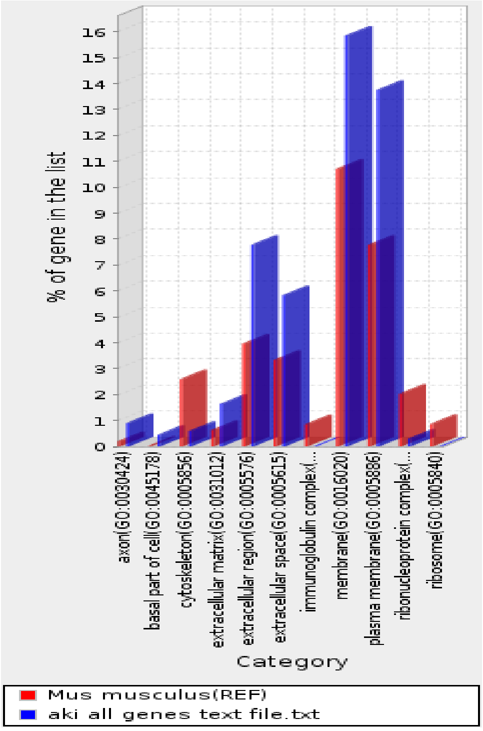

Fig C: Bar chart generated in PANTHER to compare the gene count of AKI genes with the reference mouse genes in various cellular components.
